# Bioplastic Production from Microalgae: A Review

**DOI:** 10.3390/ijerph17113842

**Published:** 2020-05-28

**Authors:** Senem Onen Cinar, Zhi Kai Chong, Mehmet Ali Kucuker, Nils Wieczorek, Ugur Cengiz, Kerstin Kuchta

**Affiliations:** 1 Sustainable Resource and Waste Management, Hamburg University of Technology, 21079 Hamburg, Germany; kai.chong@tuhh.de (Z.K.C.); nils.wieczorek@tuhh.de (N.W.); kuchta@tuhh.de (K.K.); 2 Faculty of Engineering, Department of Environmental Engineering, Canakkale Onsekiz Mart University, 17020 Canakkale, Turkey; kucuker@comu.edu.tr; 3 Faculty of Engineering, Department of Chemical Engineering, Canakkale Onsekiz Mart University, 17020 Canakkale, Turkey; ucengiz@comu.edu.tr

**Keywords:** bioplastic, microalgae, bioeconomy, biodegradable plastic, circular economy, bio-based plastic

## Abstract

Plastic waste production around the world is increasing, which leads to global plastic waste pollution. The need for an innovative solution to reduce this pollution is inevitable. Increased recycling of plastic waste alone is not a comprehensive solution. Furthermore, decreasing fossil-based plastic usage is an important aspect of sustainability. As an alternative to fossil-based plastics in the market, bio-based plastics are gaining in popularity. According to the studies conducted, products with similar performance characteristics can be obtained using biological feedstocks instead of fossil-based sources. In particular, bioplastic production from microalgae is a new opportunity to be explored and further improved. The aim of this study is to determine the current state of bioplastic production technologies from microalgae species and reveal possible optimization opportunities in the process and application areas. Therefore, the species used as resources for bioplastic production, the microalgae cultivation methods and bioplastic material production methods from microalgae were summarized.

## 1. Introduction

**Figure 1:**
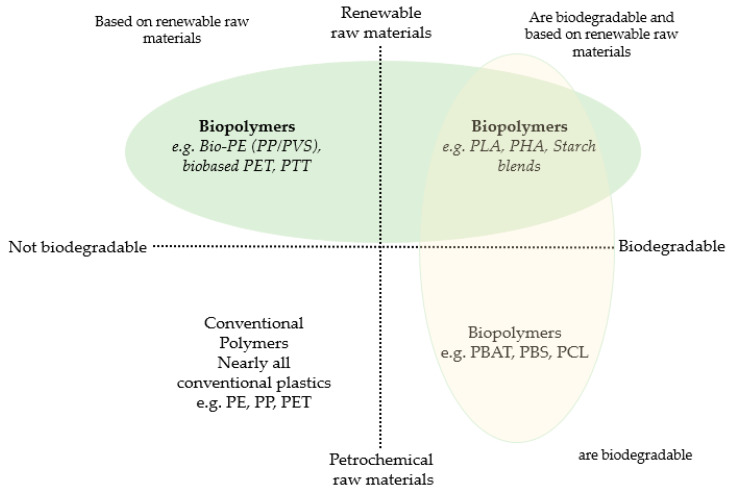
Types of bioplastics [4].

Global plastic demand, driven by the increasing usage of plastic-based materials, has been increasing and adding stress to the current waste management infrastructure [1]. There is significant interest to decrease the reliance on petroleum-based plastic products, which causes global environmental pollution [2]. More than eight million tons of plastic waste leaks into oceans every year, which can be mitigated through innovative redesigns of packaging materials [3]. In general, plastics can be classified based on two factors: fossil-based or bio-based, non-biodegradable or biodegradable. The possible combinations are presented in Figure 1 [4].

**Figure 2:**
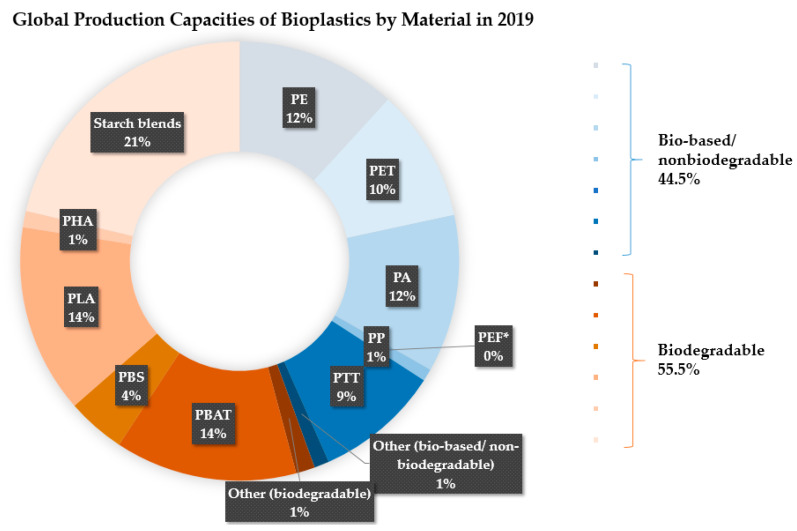
Global production capacities of bioplastics by material in 2019 [6]. PE, polyethylene; PP, polypropylene; PET, poly (ethylene terephthalate); PVC, polyvinyl chloride; PTT, poly (trimethylene terephthalate); PBAT, poly (butylene adipate-co-terephthalate); PBS, polybutylene succinate; PCL, poly(Ԑ-caprolactone) [8], PLA, polylactide; PHA, poly (hydroxyalkonate).

Bio-based plastics are further classified into three categories: Modified natural polymers, synthesized bio-based polymers from synthesized bio-based monomers and bioplastics from waste [5]. Currently, only around 1% of the annual plastic production in the world is bioplastics [6]. The share of bio-based but non-biodegradable and biodegradable plastics in the market in 2019 is presented in Figure 2. The largest share goes to starch blends, 21% of the total production in 2019. The share of bioplastics in the market is expected to increase. The main application areas are in the packaging industry, followed by the textile industry, the automotive industry and construction [6,7].

The sources that can be used for bioplastic production are plant-based raw materials, natural polymers (carbohydrates, proteins, etc.), and other small molecules (sugar, disaccharides, and fatty acids) [5]. Bioplastics production capacity increased from approximately 2 million tons in 2014 to about 6.7 million tons in 2018, where they were mostly produced from starch and poly (lactic acid) (PLA)-based polymers [5,6]. Nowadays, bioplastics are derived from terrestrial crops such as corn and potatoes and thus compete with food supplies [1]. In addition, bioplastic production from agricultural crops uses up large land areas, water, and nutrients [6,9]. Numerous studies have shown that this kind of bioplastic production is not sustainable for the long-term. Furthermore, the commercialization of bio-based compounds on a large scale has been facing many problems in the last decade [10]. Therefore, microalgae can be a potentially better biomass source for bioplastic production since it does not compete with food sources, has the ability to grow on waste resources, and can achieve high lipid accumulation [1,11,12]. In addition, bioplastic production from microalgae can be more sustainable and contributes to the circular economy as well as the bioeconomy [13,14]. Bioplastics can be used in food packaging, pharmaceuticals and cosmetics.

**Figure 3:**
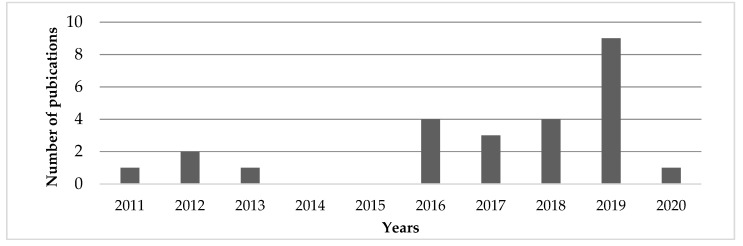
Number of publications produced between the years 2011–2020 [15].

The history of research on the production of bioplastic from microalgae was examined in the Scopus website using the keywords “bioplastic microalgae”. The number of publications published in each year was extracted. According to the results, the topic gained attention in 2016. After that, there was a spike in 2019, which shows a recent increase in interest. This is represented in Figure 3 [15]. The results of the Scopus analysis correlate to an expected increase in bio-based plastic production [7]. Another analysis in Scopus showed that most studies were conducted in the following subject areas: agricultural and biological sciences, biochemistry, genetics and molecular biology, energy and environmental sciences [15]. 

Studies on the production of bioplastic material from microalgae sources can be grouped into two main approaches. Bioplastic can refer to composites produced by blending microalgae biomass, bio- or petroleum-based polymers and additives. These products are produced through thermal-mechanical methods such as compression molding. The other approach is based on the cultivation of biopolymers such as polyhydroxybutyrates (PHBs) and starch intracellularly within microalgae cells. These products can be then extracted and further processed for bioplastic production. In this case, the microalgae cells are not utilized directly. 

Research activities conducted include: (1) blend design of microalgae-polymer composites, (2) processing of microalgal bioplastic in a biorefinery context, and (3) genetic engineering to create biopolymer producing strains of microalgae [1]. However, there is still a need to improve the technologies and their efficiencies to enable commercialization, industrialization and scale-up. In this study, numerous microalgae species used in bioplastic production are reviewed in order to find research gaps surrounding the topic. Then, the cultivation opportunities of microalgae including medium types are discussed. Lastly, this review paper evaluates the state of the field with regards to production conditions and methodologies.

## 2. Microalgae and Additives

**Figure 4:**
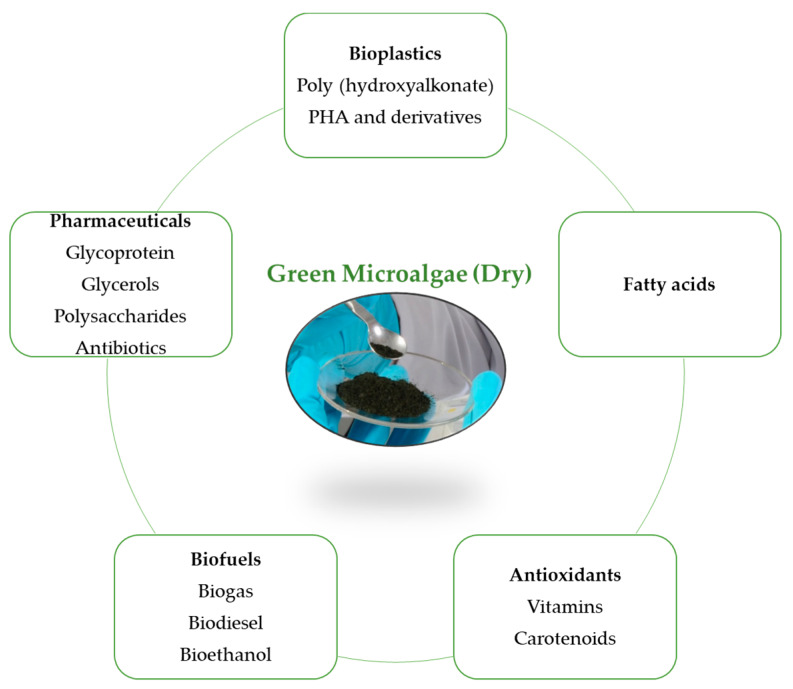
Common products of microalgae [18,19].

Algae species are classified into macroalgae and microalgae. Collectively they include more than one million species. Microalgae are microscopic organisms, which utilize solar energy to produce adenosine triphosphate (ATP) and live in freshwater and marine environments [16]. Microalgae can replace many other sources for biofuel production, be used as supplements in the food industry, cosmetics and also in pharmaceutical formulations. Microalgae is becoming increasingly popular due to its potential contribution to the bio-economy [17]. The common microalgae products are shown in Figure 4.

The main components of microalgae are lipids (7–23%), carbohydrates (5–23%), and proteins (6–52%) [20]. In addition, microalgae contains calcium (0.1–3.0%), magnesium (0.3–0.7%), phosphorous (0.7–1.5%), potassium (0.7–2.4%), sodium (0.8–2.7%), sulfur (0.4–1.4%), copper (18–102 mg·kg^−1^), iron (1395–11,101 mg·kg^−1^), manganese (45–454 mg·kg^−1^), selenium (0–0.5 mg·kg^−1^), and zinc (28–64 mg·kg^−1^) [21]. Commonly used microalgae species, additives or chemicals utilized in bioplastic production will be explained in the following sections. 

### 2.1. Chlorella

*Chlorella* is a genus of green algae, which can be found in freshwater and contains around 58% (by weight) protein. It has a higher crack resistance due to its dense cell walls and higher thermal stability compared to *Spirulina* [9,22]. This species is often used in biomass–polymer blends. According to Zeller et al. (2013), after comparing bioplastic production from 100% microalgae biomass and blends containing additives and polymers, it was found that blending is necessary for commercial applications [9]. Tests conducted to measure product quality showed that higher quality bioplastic could be obtained using *Chlorella vulgaris* compared to *Spirulina*. However, *Spirulina* has better blending properties compared to *C. vulgaris* based on the physicochemical features of the material [9]. Another study was conducted by Dianursanti (2018) to examine the effect of the compatibilizer ratio on the quality of produced PVA (polyvinyl alcohol)-*C. vulgaris* composites. The study indicated that the best quality can be obtained with a compatibilizer (maleic anhydride) concentration of 6% in the mixture [23]. Starch granules (1 µm) can also be produced from *C. sorokiniana* microalgae biomass. Starch is a significant biopolymer used in the food, chemical and bioplastic industries. Due to its high gelatinization temperature (110 °C), it is attractive for starch-based bioplastic production [24]. Zhang et al. (2000) found that hydrogen ions present among *Chlorella* cells allow blends to be produced without gaps [25]. The implementation of ultra-sonic homogenization pre-treatment before blending can improve the homogeneity and surface features of the produced *Chlorella*–PVA blends, which is an alternative for food packaging [26]. The difference between *Chlorella*–PE composites with and without modification of PE (with maleic anhydride) was examined by Otsuki et al. Modification of PE positively affected the tensile strength of the composites [27]. The studies conducted with *Chlorella*, *C. vulgaris*, *C.* sp. are summarized in Table 1.

### 2.2. Spirulina

*Spirulina*, which is used for many years in the food industry as a protein source, is known for its adaptation potential to extreme environments [28]. *Spirulina platensis* contains a high concentration of protein. Its composition is shown in Table 2 [29]. 

There have been several studies conducted to examine the potential of *Spirulina* for bioplastic production (Table 3). Similar to *Chlorella, Spirulina* has a small cell size, which makes both of them attractive for bioplastic blend production. Despite their similarities, *Chlorella* and *Spirulina* showed different behaviors and bioplastic properties while blending with PE due to their varying amino acid contents. The addition of compatibilizers can improve the product properties of *Chlorella*-based bioplastics [9]. With the addition of 6 wt % of a compatibilizer to an *S. platensis* and PVA mixture (Table 3), a bioplastic film which had higher tensile strength than commercial plastic bags was produced. The usage of a compatibilizer also increased the elongation capability of the plastic and enabled smoother surfaces [29]. In a study conducted by Ciapponi (2019), *S. platensis* was used as a reinforcement material in plasticized wheat gluten. Nevertheless, the microalgae biomass particles which have particle diameters higher than 5 µm did not show efficient reinforcement ability. The smaller particles were able to blend with the other materials more efficiently [30]. To increase the flexibility of the plastic produced from *S. platensis*, glycerol was added at different concentrations (15–30%). Higher tensile strengths and lower elongations were observed from the produced bioplastic with the addition of 30% glycerol compared to commercial plastic bags. These results showed that the produced bioplastic can be used for food packaging, in pharmaceutical applications or cosmetics, where high elongation is not needed [22]. Wang (2014) reported the differences between a blend of *Spirulina* with an ultra-high molecular weight PE (UHMW)-PE and compatibilized *Spirulina* (with ethylene glycol) with maleic acid-modified PE (PE-g-MA) [31]. Homogenous phase distribution and high inter-surface adhesion can be obtained after the plasticization of *Spirulina*. Improvements in the homogeneity and tensile properties are also possible after the implementation of blending. On the other hand, the addition of compatibilizer did not improve the mechanical properties of the produced bioplastic [31]. The addition of maleic anhydride-grafted PBS to biomass as a compatibilizer improves the tensile strength of the product and decreases degradation temperature as well [32].

### 2.3. Other Microalgae Species Used for Bioplastic Production

As mentioned in the sections above, most of the studies in this field were conducted to examine bioplastic production from *Chlorella* and *Spirulina*. In addition to these studies, there has been some research conducted using other microalgae species. These are summarized in Table 4. 

According to Monshupanee et al. (2016), who studied intracellular production of PHB using microalgae, PHB (polyhydroxybutyrate) accumulation during the production process can be optimized using the amount of acetate supply, varying light and nutrient conditions [33]. Optimum production rates could be obtained by implementing heterotrophy in the dark [31]. PHB levels of 10.6% to 17% of dry weight can be obtained respectively from *Chlorella fritschii* and *Phaeodactylum tricornutum* [11,31,33]. *Calothrix scytonemicola, Scenedesmus almeriensis,* and *Neochloris oleobundans* are also suitable for intracellular biopolymer production since they are either starch or PHA rich microalgae species. 

For biocomposites, species studied include *Nannocloropsis gaditana,* which contain 23% protein, 33% fat, 22% ash, 22% fiber and free carbohydrates in the biomass. Injection-molded and extruded biocomposites were also prepared from residual microalgae biomass (RMB) and PBAT with various ratios of RMB (10, 20, and 30). According to tests conducted after the production, the best results were obtained with 20% RMB, which were produced with the extrusion method [34]. 

### 2.4. Additives Used in the Production Process

#### 2.4.1. Materials Blended with Microalgae Biomass

Conventional polymers are commonly utilized to produce blended bioplastics from microalgae to achieve improved plastic properties [9,31,36]. Table 5 summarizes the research done in this area. PE and PP are the most commonly used polymers in the blending process. They represent more than two-thirds of world plastic demand [37]. PE is consumed in a wide variety of industries such as cosmetics, food packaging, and medical products including prosthetics [38]. UHMW-PE is an extremely long chain PE that has a molecular weight between 2 and 6 million. Because it is non-toxic and odorless with extremely low moisture absorption capacity, it is preferable for bioplastic production [39,40]. In the study conducted by Wang (2014), UHMW-PE at different ratios (20–80% at 15% intervals) were blended with *Spirulina*. Better tensile strengths were observed at the ratio 80:13:7 of PE-*Spirulina*-EG (Ethylene Glycol) [31].

On the other hand, because PP has a semi-transparent look and is strong against heat and mechanical effects, it is preferred for the production of packaging for yogurt, medicine, and beverages [41]. PVA was used in a study to produce bioplastic films with *Chlorella* at selected solution temperatures. Higher temperatures were found to lead to weaker bonds between blended materials. For blends, the implementation of ultra-sonication improved the quality of the produced blended material due to its effect on the homogeneity of the mixture [26]. 

Wheat gluten is widely studied to produce sustainable bioplastic. Although, it has a brittle structure, the structure of the material can be improved with additives and fillers. Because of wheat gluten´s high protein content, it is promising for many application areas [30,42]. PBS is defined as one of the newest biopolymers by Sisti (2016), which could meet increased bioplastic demand in the market. PBS is usually preferable compared to low-density polyethylene (LDPE) and PP [43]. Biodegradable PBS can be produced from biomass or fossil-based sources. PBS is especially utilized in the textile industry due to its easy processability. For example, it is used for the production of melt blow, multifilament, monofilament, flat and split yarn. Moreover, it is used in the plastic industry to produce molded products [44]. Blending PBS with other polymers improves its mechanical properties to increase its application range [45]. Blending PBS with *Spirulina* allows economically efficient production of *Spirulina*-based bioplastic [32]. 

Several chemicals were used in studies to improve the process efficiency of blending and product quality, as represented in Table 5. PVA is known for its ability to increase the strength, durability, and flexibility of the product. On the other hand, the modification of PVA with MA is necessary to improve the surface and dimensional stability as well as the mechanical properties of packaging materials [22,23]. Acetone was used to wash biomass to achieve small spherical grains suitable for blending [27,35,46]. In bioplastic production, sodium sulfite was also used for biomass washing before blending it with other materials [32]. In the production process of *Chlorella*-PP composite, benzoyl peroxide (BPO) was mixed with acetone and MA to be sprayed on PE powder to improve blending properties [27]. 

#### 2.4.2. Plasticizers and Compatibilizers

Plasticizers are bulky organic molecules, which are mixed with materials to improve their flexibility and processability. The efficiency of the plasticizer is related to its ability to make the target material softer [47,48,49]. Glycerol (C_3_H_8_O_3_) is the most used plasticizer in bioplastic production using microalgae as shown in Table 6 [22,29,30,32,50]. In the studies conducted, glycerol improved the availability of the macromolecules for the degradation process [9], increased flexibility and extensibility [29], led to phase rich products and improved elongation [22]. The plasticization abilities of glycerol, octanoic acid and 1,4-butanediol with wheat gluten and microalgae as filler were compared in the study conducted by Ciapponi et al. (2019). After the implementation of various tests, it was found that glycerol and 1,4-butanediol can be implemented in the plasticization process efficiently based on its permeability against water [30]. To improve the mechanical properties of the produced plastic, carboxymethylcellulose (CMC) was also implemented [35]. CMC is an end product of the reaction between cellulose, alkali and chloroacetic acid, which dissolves easily in cold water, has low viscosity and is thermally stable [51].

Compatibilizers, which consist of two parts; one part is compatible with one polymer and the other part is compatible with the other target polymer, are used to bind two polymers [52]. The compatibilization process increased the mechanical strength of the heterogeneous biopolymers [53]. For different kinds of blends, various compatibilizers can be used, such as maleic anhydride, grafted ethylene/propylene rubber, poly(ethylene-co-glycidyl) meth acryloyl carbamate, and diethyl succinate [52]. MA was used at different concentrations in the study conducted by Dianursanti (2018). The addition of maleic anhydride increased the homogeneity and flexibility of the products [22]. In another study conducted by Zhang et al. (2000), MA was grafted with PE by creating ester bonds between MA and PE, and enabled bonding between hydroxyl groups on the cell walls of *Chlorella* and PE [25]. PE-g-MA was added to UHMW-PE and *Spirulina* composites in eight different percentages increasing by 3%. The addition did not have a significant effect on the dynamic mechanical properties of the composite [31]. In addition to mentioned plasticizers and compatibilizers, potassium peroxide sulfate (KPS) and dimethyl sulfoxide (DMSO) were used as compatibilizer initiator. The process was as follows: before the addition of KPS (1% of PVA), DMSO (15 mL), MA, and PVA were melted [22].

#### 2.4.3. Other Chemicals Used in the Process

The chemicals used in the isolation, blending, or plasticization process to improve the quality of the composites is summarized in Table 7.

## 3. Cultivation of Microalgae

Several microalgae cultivation methods were described in literature, the primary aim of which is to produce bio-components for the production of biomaterials [54,55,56,57]. The cultivation of microalgae exclusively for the production of food and bioenergy sources do not currently seem feasible due to reduced economic attractiveness [58]. To develop more sustainable and economically feasible processes, the production of primary components such as proteins, saccharides and lipids, together with secondary components such as bioplastics, pigments, antioxidants or vitamins should be considered [59]. These components can be used for the production of food and animal feed, cosmetic and pharmaceutical preparations, as an antioxidant, and energetically for the production of biofuels. In addition, microalgae can synthesize essential organic substances such as chromophores, vitamins and fatty or amino acids in high concentrations, which is why they are now considered a potential raw material supplier for future industrial processes. The type and amount of the synthesized substances are determined not only by the organism itself but rather by factors of chemical, physical and biological origin. These factors can be regulated in a targeted manner and consequently influence the synthesis of the cell contents and the growth. If cultivation takes place outdoors, these influencing factors are always in direct interaction with external factors such as day-night cycles or seasonal changes. 

The available systems for the cultivation of microalgae have distinct disadvantages and advantage each. Thus, the optimal system cannot be determined at the moment. For example, Mata et al. (2010) reported that open cultivation systems have lower investment costs as well as higher production capacities when compared to closed cultivation systems [60]. Whereas Singh and Sharma (2012) highlighted better process control and the lower risk of contamination of closed systems [61]. Milledge et al., (2011) [62] described separators as the most efficient system for harvesting algae, whereas Knuckey et al. (2006) [63] indicated possible cell damage due to gravitational and shear forces. The following section, therefore, describes the most important systems for cultivating microalgae, their preparation and discusses their potential applicability.

### 3.1. Production Systems

**Figure 5:**
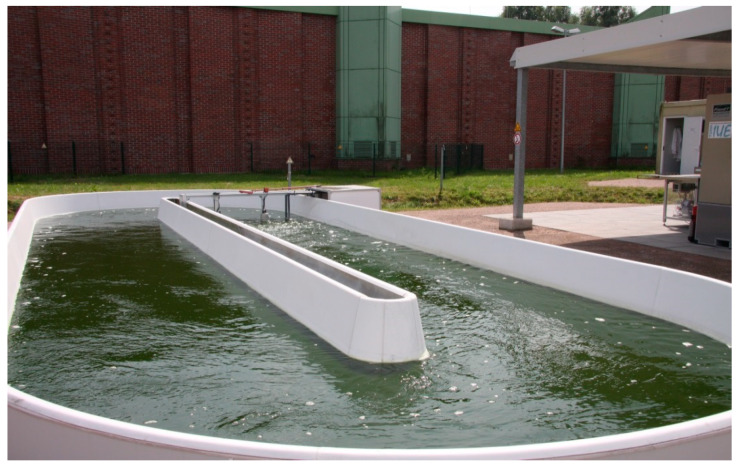
Open pond cultivation system.

**Figure 6:**
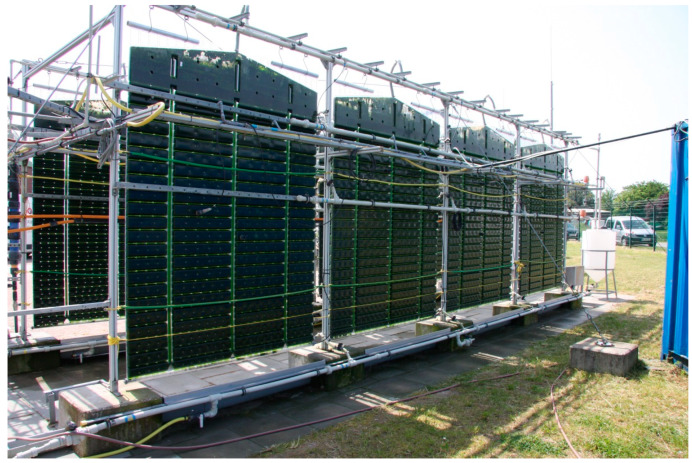
**Flat panel** photobioreactors (PBRs) cultivation system.

Numerous systems for microalgae cultivation are described in the literature, which differ fundamentally from one another in terms of construction, production material, and process control [60,64,65,66]. According to Ugwu et al. (2008), despite a large number of known systems, limited systems are suitable for cultivation on an industrial scale [67]. The open systems such as raceway ponds are the primary cultivation systems, the closed systems include photobioreactors (PBR). Examples of open and closed systems are shown in Figure 5 and Figure 6, respectively.

A summary of the advantages and disadvantages of open and closed PBR systems is shown in Table 8. Comparing the two systems, open ponds are primarily characterized by a higher cultivation or production volume compared to photobioreactors. In addition, open systems are considered easy to clean due to their simple design and generally more durable. However, the decisive advantage is mostly the economic attractiveness both in the production and in the cultivation company [60,67]. In addition, according to Harun et al., (2009) the production site is a decisive criterion for choosing a suitable system [66]. Regions with intensive light exposure are particularly advantageous when using open systems. However, it must be taken into account here that intensive exposure to light usually brings a high temperature with it and consequently water loss, because evaporation must be taken into account. The limited control possibilities of cultivation parameters such as temperature or pH are also listed as a disadvantage. In addition, the algae cultures used in open systems are generally not actively supplied with CO_2_ and consequently, only low cell densities and productivity can be achieved. According to Vonshak (1997) and Pulz (2001), the cell density in open systems is of the order of about 0.1 to 0.5 g·L^−1^ [68,69]. For productivity, Richmond (1990) and Pulz (2001) generally describe orders of the magnitude of approximately from 0.06 to 0.1 g·L^−1^·d^−1^ and 10 to 25 g·m^−2^·d^−1^, respectively [69,70]. However, the decisive disadvantage of open systems is the high risk of contamination with bacteria or foreign species, which severely limits the potential usability of the microalgae [60,67,71]. According to Singh and Sharma (2012), the commercial production of microalgae in open culture systems is restricted to those species that can grow under extreme conditions [61].

Closed PBR systems can generally be divided into three categories: horizontal tube PBRs, vertical tube PBRs and flat plate PBRs. Horizontal tubes PBRs generally have the advantage of a large surface area, so that high cell densities and productivity can be achieved in these systems. On the other hand, it is described as a disadvantage that the length of the pipe systems can lead to a gradient in the pH value and in CO_2_ as well as O_2_, which inhibits the growth of the microalgae. Compared to open systems, cleaning is also more complex and the cultivation or production volume is lower [60,67]. Due to the design, vertical tube PBRs have suitable mixing and a high level of exchange. This kind of reactor can also be sterilized and tempered. One drawback of vertical tube PBRs is small surface areas that cause high shear stress for the algal cultures [60,67]. On the other hand, flat-panel PBRs have the largest surface area and a short light distance.

This combination enables significantly higher cell densities and productivities to be achieved in flat-panel PBRs than with the systems described above. Another advantage is that the reactors can be cleaned and tempered relatively easily. Due to the design, culture can be mixed well. The design also ensures fine material exchange and prevents gradients from forming. A drawback of these reactors is that a large number of individual compartments is required for a scale-up. The use of separate compartments also makes process control and it is more difficult, since each reactor has to be operated individually [60,66,67]. In summary, it can be stated that PBR systems have several advantages than open pond systems. Depending on the construction and design, PBR systems have better control of culture conditions such as pH, temperature, material exchange, prevent evaporation, offer a safer and protected environment, prevent the entry of foreign substances and minimize the risk of contamination by competing microorganisms [60,61]. Despite these advantages, Mata et al., (2010) describe several challenges that must be solved before they can be used in a large-scale application [60]. These primarily include the rapid overheating of the algal culture, the formation of biofilms, difficulties with scale-up and the high investment and operating costs. The information about what productivity levels and cell densities can be achieved in PBR systems is extremely different. Doucha et al. (2005) [72] and Carlozzi (2003) [73] reported that the productivities of over 3 g·L^−1^·d^−1^. Olaizola (2000) [74] and Sato et al. (2006) [75], however, reported productivities only in the order of about 0.05 to 0.09 g·L^−1^·d^−1^. Cultivation ranges between 25 and 50 g·m^−2^·d^−1^ in several studies [60,76,77]. The typical cell density in PBR systems is in the order of about 2 to 6 g·L^−1^ and can be increased up to 20 g·L^−1^ [65,78].

### 3.2. Harvesting and Processing Systems

A variety of processes and systems are available for harvesting and processing microalgae. The selection of a suitable method can be of central importance in the development of a bio-series since the microalgae harvest alone can account for between 20% and 30% of production costs according to Rawat et al. (2011) and Salim et al. (2011) [79,80]. It should be primarily determined which type of harvesting technique is the most effective for the type of microalgae. In this context, the most important criteria include morphological features such as cell size, density or specific surface charges [81,82]. According to Uduman et al., (2010) and Chen et al., (2011), the most established harvesting methods involve separation, centrifugation, flocculation, filtration, sieving, sedimentation and flotation [54,81]. Methods such as filtration or sieving can be ruled out for harvesting of some microalgae due to the small cell size. The algae harvest using a separator or centrifuge is considered the most efficient of the methods listed in the literature [62]. Additionally, the use of separators offers the possibility of continuous operation and, in comparison to sedimentation or flotation, the use of high throughput quantities. According to Knuckey et al., (2006), the disadvantage of this method is the high gravitational and shear forces, which can damage the algae cells [63]. The decisive drawbacks are the energy costs associated with this process, which often make the use appear unattractive [81]. Sedimentation is an inexpensive alternative to algae harvesting through separation. In sedimentation, the algae cells are separated from the culture medium based on the weight. However, the efficiency of this method is largely determined by the size and density of the cells [54]. To increase the efficiency of sedimentation of microalgae, their flocculation properties can be promoted in various ways. This includes, for example, adding chemical flocculants or changing cultivation parameters, such as increasing the pH in the culture medium [76]. However, the use of flocculants or adjusting the pH can have disadvantages in the context of biomaterials. This is the case when the separated culture medium is to be recycled after the algae harvest and returned to the reactors. Another possibility, which increases the efficiency of the harvest and at the same time reduces the costs, is the combination of different processes. Here, a two-stage process appears, in which an enrichment first and then the drainage takes place on the promising one. The combination of sedimentation and separation offers an attractive option [83].

## 4. Bioplastic Production Technologies

Generally, current production techniques for microalgae bio-composites are still lab-scale and focused on the feasibility of blend design. In the second approach, where biopolymers are produced intracellularly or by further processing biomass, researchers focused on genetically optimizing the biopolymer producing strain, improving cultivation plant designs and strategies.

### 4.1. Production of Microalgae-Polymer Blends

The most common route to produce microalgae-polymer blends is through compression molding, where a mixture of biomass, polymers and additives are placed in a mold and compressed at elevated pressure and temperature for a short duration to form bio-composites. The temperature, pressure and time parameters vary significantly in current literature. The reported temperatures used range from 130 to 160 °C, compression pressures range from 20 kPa to 10 MPa while the molding time ranges from 3 to 20 min. 

Before the molding process, the mixtures need to be uniformly mixed. Some publications employ heating during this step and conduct what is called melt mixing. Similar to compression molding, the parameters used during this step is not standardized and depend on the research group. Fabra et al. (2018) utilized an internal mixer from Brabender designed for material research to melt mix their blends at 130 °C and 60 rpm for 4 min before compression molding [84]. On the other hand, Otsuki et al. (2004) used a simpler roller mixer for melt mixing at 160 °C for 7.5 min. Before blend homogenization, they also utilized the same process to produce MA-modified PE to facilitate the binding of microalgae biomass to the polymer material [27]. Besides compression molding, some studies adapted variations excluding the pressure element. The method used by Dianursanti et al. (2018) involved heating the melt mixed mixture in molds in ovens without compression to form their prototypes [22,23]. 

The molding techniques produced prototypes of different shapes and sizes, depending on the dimensions of the mold used. The prototypes can be in the form of films [84], slabs [30], dog bones, or rectangular flex bars [9]. The purpose of the study determines the shapes needed. For example, Fabra et al. [84] were interested in developing biodegradable packaging while Zeller et al. (2013) [9] have a wider focus in terms of product application including horticultural products.

Solvent casting is another route of prototype production used for films. Here, biomass, polymers and additives in a formulation are dissolved in a solvent, cast on surfaces and dried to form films. The parameters also vary from publication to publication. Sabathini et al. (2018) produced microalgae-PVA films by dispersing the components in water, casting the mixture onto glass plates and air drying 24 h [26]. An extra homogenization step is conducted by Zhang et al. (2000) on the biomass aqueous mixture before the addition of the polymer suspension to ensure proper dispersion of the biomass particles [85].

The majority of the publications focus on testing the feasibility of blend design and optimizing the concentration of the components. The production methods employed are often lab scale. More scalable methods such as injection molding and twin-screw extrusion were however also implemented. To prepare biomass-PBAT prototypes, Torres et al. (2015) used a combination of a micro twin-screw extrusion at 140 °C, 100 rpm for 2 min and subsequent injection molding at 30 °C [34].

The parameters are typically not varied within each study since the main research focus is usually on optimizing the component concentration within the mixture. For example, Ciapponi et al. (2019) studied the effects of composition on the tensile strength, water permeability and thermal stability of the resulting material [30]. Table 9 summarizes the production methods employed to date and the relevant publications. Compression molding and solvent casting are the two most used techniques.

#### Testing Methods Used for Blend Characterization and Performance Measurement

After prototype production, tests are conducted on the material to determine performance and to investigate its structural and molecular properties. The optimal properties depend highly on the potential application of the product and also the type of material used for the composite. Table 10 shows a summary of the testing methods employed and the relevant publications. It shows that mechanical testing, thermal based analytical methods, SEM and FT-IR are the common methods used to assess the performance and characterize the prototypes.

The mechanical properties are one of the core performance indicators chosen by studies on microalgae–polymer blends. Mechanical properties frequently measured are tensile strength and elongation at break and they are often compared between varying blend compositions. To this end, standards for mechanical testing such as ASTM D412 [88], ASTM D630-10 [30], ASTM D638 [34] and ASTM D882-12 [22,26,32,84] were quoted. Otsuki et al. (2004) found that the tensile strength and elongation at break (%) decreases with increasing biomass content [27]. Similarly, Zhang (2000) et al. also reported that the tensile strength of the PVS/*Chlorella* composite materials decreases by increasing the *Chlorella* content [89]. Conversely, Ciapponi et al. (2019) observed an increase in tensile strength with increasing biomass concentration [30]. The interactions between blend components are complex and highly dependent on the type of biomass, polymer and additives utilized. While this additive increases some properties such as elasticity and adhesion between glass and composite materials of the blends, the mechanical properties were decreased. Dianursanti et al. (2018) [22] investigated the tensile strength of PVA-g-MA/*Chlorella* composite films MA as a compatibilizer. They reported the highest tensile strength as non-MA composite films. 

Other tests that directly inform the prototypes’ performance are water and oxygen permeability, which are critical properties if the material is to function as packaging. For example, Fabra et al. conducted water vapor permeability tests using Payne permeability cups according to the ASTM E96/E96M-10 standard. Oxygen permeability was also inferred from measurements of the oxygen transmission rate [84]. They found that water permeability dropped while oxygen permeability increased with the addition of biomass to the corn starch film matrix. In addition to the water vapor transmission rate, Ciapponi et al. (2019) also additionally measured the liquid contact angle and carried out kinetic water absorption tests [30]. They found that the addition of biomass increased surface sensitivity to water but decreased the overall water absorption kinetics. In terms of performance, Wang et al. (2014) conducted odor tests to evaluate the effects of activated carbon and zeolite additives [86].

Thermal based analysis such as thermogravimetric analysis (TGA) and differential scanning calorimetry (DSC) is commonly used to determine the thermal stability and analyze the composition of the prototypes. By comparing TGA curves for blends with increasing biomass content, Ciapponi et al. (2019) and Torres et al. (2015) observed a slight increase in thermal stability of the composite [30,34]. Zhu et al. measured and compared the crystalline temperatures and degree of crystallinity of blends with varying compositions using DSC curves [32]. Lastly, Zeller et al. (2013) analyzed DSC curves to intermolecular effects within the blends [9].

Scanning electron microscopy (SEM) is a popular technique to observe the morphology of the microalgae-polymer composites formed within the studies reviewed. In particular, the interactions between different components in the composite are of interest. Observing the fractures on the prototype after a stress-strain test, Otsuki et al. (2004) found that the interaction between the biomass and polymer material is stronger than within the biomass material itself [27]. Fabra et al. in turn observed the cross-section of the biocomposite films produced and concluded that the microalgae cells remained intact even after melt mixing [84]. Besides, the group also measured the transparency of the material using a Spectrocolorimeter. Torres et al. (2015) used confocal laser scanning microscopy (CLSM) to highlight biomass dispersion within the composite [34].

Fourier transform infrared (FT-IR) spectroscopy is used to provide information on the chemical bonds within the composites and allow the comparison of any bond changes after processing. Zhang et al. compared the FT-IR spectra of composites produced in both an alkali and acidic environment. They attributed the increased elongation ability of the composite formed in an acidic environment to more hydrogen bonds formed as indicated by FT-IR analysis [85]. Besides FT-IR analysis, other less common spectroscopic methods deployed include wide-angle x-ray scattering (WAXS) and small-angle x-ray scattering (SAXS) by Fabra et al. to assess the crystalline structure and layers of the prototypes [84]. 

### 4.2. Production of Biopolymers Using Microalgae Cells

Alternative routes to bioplastic products from microalgae sources are through the production of biopolymers intracellularly in microalgae or fermentation using microalgae biomass. In most intracellular production methods, PHB is the focus as it is already naturally synthesized by a variety of microbes.

Some researchers conducted genetic engineering experiments to enable or increase the production of PHB in microalgae species. Onasai et al. (2013) grew a strain of *Synechocystis sp.* that overexpresses the sigma factor sigE. They confirmed the increased production of PHB compared to the control during nitrogen starvation [91]. Hempel et al. (2011) introduced the bacterial PHB pathway into the microalgae species *Phaeodactylum tricornutum* and reported the production of PHB up to 10.6% microalgae dry weight [11]. 

Other groups focused more on cultivation methods and parameters. Troschl (2018) tested a pilot-scale photobioreactor using *Synechocystis sp.* A two-stage cultivation strategy was utilized where growth is prioritized during the first stage with available nutrients and PHB production is induced in the second stage when the nutrients are depleted [92]. Monshupanee et al. (2016) also employed a two-stage cultivation strategy, first phototrophic followed by heterotrophic, of cyanobacterium for PHB production [33]. 

Biorefinery concepts that include PHB production are also studied to reduce the overall cost of the products by leveraging the simultaneous production of multiple products. Das et al. (2018) chemically treated leftover microalgae biomass after biodiesel production and extraction to produce PHB based bioplastic material [93]. Uggetti et al. (2018) in turn tried to selectively cultivate PHB producing cyanobacteria by controlling the nutrient input. The main feedstock is wastewater and agricultural runoffs [94].

For more detailed information, refer to the reviews published focused on PHB production using microalgae [95,96,97]. In addition to PHB, there are also notable publications studying the production of starch through microalgae [24,35,98,99].

**Figure 7:**
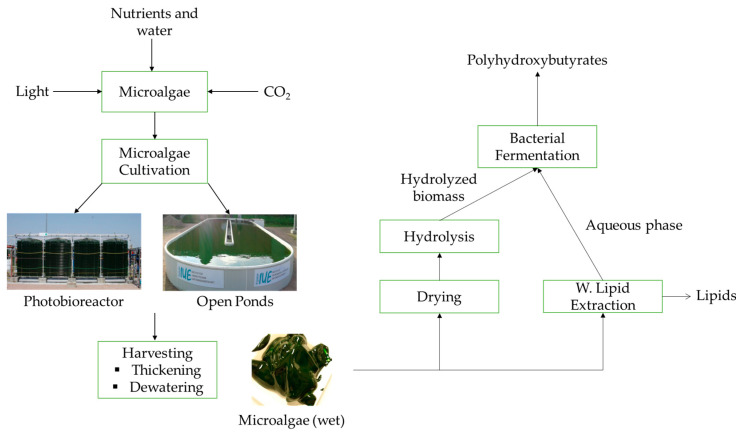
Bioplastic production from microalgae [1].

The bioplastic production process from the microalgae biomass is shown in Figure 7. The first step is cultivation followed by harvesting [100]. The cultivation step is mainly performed with open ponds or photobioreactors (PBRs). The different types of PBRs include flat-plate, tubular, vertical column, and airlift [101]. Harvesting is the fundamental step to collect the microalgae biomass. The techniques include filtration, flotation centrifugation, and sedimentation [102]. The most suitable method is flocculation due to its low energy requirement [100]. A universal cultivation and harvesting method is not recommendable taking into account the varying characteristics of different microalgae. The optimum method should be selected based on the microalgae types and the final product needed [1,100]. 

After cultivation and harvesting, two different methods can be highlighted to produce PHB from the microalgae illustrated in Figure 7. The first method is the hydrolysis of microalgae biomass after drying [103]. In this method the dried microalgae is hydrolyzed to produce fermentable sugars [104]. After the hydrolyzed stage the biomass could be used for *Esherichia coli* fermentation and production of PHB [1,105]. The second method is the wet lipid extraction procedure (WLEP) and this method has the potential to reduce the overall cost of the system not to use the drying step [103].

## 5. LCA Studies on Bioplastic Production from Microalgae

There are not many LCA (Life Cycle Assessment) studies published specifically for bioplastics produced from microalgae. Bussa et al. (2019) compared PLA production from microalgae and plant-based sources and found significant environmental impact improvement potential in terms of land use and terrestrial ecotoxicity through the microalgae route [106]. In another study, Beckstrom (2019) compared the greenhouse gas intensities of different microalgae cultivation systems for bioplastic production and found better impact values for cyclic flow photobioreactors compared to open raceway ponds and combined systems [107]. The results of this study do not however provide insights on the performance of microalgae based bioplastic compared to conventional alternatives.

However, some trends can be hypothesized from LCA studies on microalgae cultivation in general. Medeiros et al. (2015) found that the fossil fuels performed slightly better compared to biofuels produced from microalgae in the LCA studies reviewed. They noted however that microalgal production systems have a much high improvement potential in terms of greenhouse gas emissions compared to fossil fuels [108]. LCAs on biofuel production from microalgae often suffer from data uncertainty and vary greatly in their results [108,109]. For food commodity production, Draaisma et al. (2013) found that microalgae based production performs well in terms of land use but less so in other impact categories such as freshwater demand [110].

In general, the environmental benefits of microalgae based production are inconclusive, though researchers often note the improvement potential of microalgal production systems. Synergies for example could be achieved through biorefineries producing multiple products and the optimization of cultivation techniques. Potential improvements to the overall LCA scores by utilizing microalgal waste for bioplastic production are also conceivable. Currently, microalgae production systems mainly shine in terms of reduced land usage.

## 6. Conclusions

In this study, the current situation of bioplastic production from microalgae resources was examined. *Chlorella* and *Spirulina* species were the most commonly used in the production of both biopolymers and plastic blends. Additives such as plasticizers, compatibilizers, and various chemicals were used as blending material to increase the quality of the final product. According to the conducted literature review in this study, there is still need for more development of bioplastic production processes from microalgae to overcome the economic feasibility problems in industrial-scale implementations, which prevents wider usage of bioplastic products from microalgae in the market. Here, a biorefinery concept where bioplastic is produced from by-products of high value chemical production from microalgae is promising. Moreover, the use of various additives may restrict the implementation areas of the microalgae products, such as in food packaging and health care. Therefore, further research is necessary to optimize the process for industrial applications and decrease the additive usage by more innovative design. 

## Figures and Tables

**Table 1:** Summary of conducted studies: *Chlorella*, *C. vulgaris*, *C. sorokiniana* and *C.* sp.

Biomass Species	Type of Product	Ratio of Materials	Particle Size of Biomass	Characterization	Reference
*C. vulgaris*	100% algae-based plastics and hybrid blends with PE and glycerol	Glycerol 0–30% (by weight)	53–75 µm	57% protein	[9]
*C. vulgaris*	*Chlorella*/PVA composites	Compatibilizer (MA) concentration 0–6%	-	58.5% protein (on the dry weight basis)	[22,23]
*C. sorokiniana*	Starch granules	-	-	38% starch (on the dry weight basis)	[24]
*Chlorella*	PP from *Chlorella* and MPP (maleic anhydride-modified polypropylene)	MPP/*Chlorella*: 0.5	50 µm	-	[25]
*Chlorella*	*Chlorella*/PVC composites	Stabilizer (PSE-227 and KM-55) concentration 0–2.5%	62–66 µm	-	[25]
*Chlorella*	*Chlorella*/PVA blend film	*Chlorella*/distilled water: 2.8 g/8.4, 14, 28 mL	-	-	[26]
*C.* sp.	*Chlorella*/PE composites	*Chlorella*/MPE (modified PE):10–40% *Chlorella*/UPE (unmodified PE):10–40%	~1 mm	-	[27]

**Table 2:** Composition of *Spirulina platensis* [29].

Component	(wt %)
Protein	60
Lipid	6
Fatty acid	265 mg·10 g^−1^
Amino acid	2410 mg·10 g^−1^
Vitamin A	2300 IU
Vitamin B1-B3	2.3 mg·10 g^−1^
Vitamin B6 & B12	112 mcg
Vitamin E	4 IU
Phycocyanin	20%
Chlorophyll	1.5%
B-Carotenoids	0.15%
Pantothenic acid	4 mg·100 g^−1^
Folic acid	100 mg·100 g^−1^
Polysaccharide	0.4 g·100 g^−1^

**Table 3:** Summary of conducted studies: *Spirulina* and *S. plantensis*.

Biomass Species	Type of Product	Ratio of Materials	Particle Size of Biomass	Characterization	Reference
*S. platensis*	100% algae-based plastics Hybrid blends with PE and glycerol	Glycerol 0–30% (by weight)	53–75 µm	57% protein	[9]
*S. platensis*	Bioplastic biofilm	Compatibilizer concentartion: 0–6%	-	60% protein	[29]
*S. platensis*	Bio filler	10%, 20% and 30% microalgae	-	-	[30]
*S. platensis*	*S. platensis*-based plastic	Plasticizer concentrations: 15%, 20%, 25% and 30%	-	-	[22]
*Spirulina*	Plasticized *spirulina*	EG weight rations: 5%, 10%, 30%	-	-	[31]
*Spirulina*	Blend of *Spirulina* with UHMW-PE	PE weight ratios: 15%, 20% and 80%	-	-	[31]
*Spirulina*	Compatibilized bioplastic	weight percentage of 3% compatibilizer (PE-g-MA)	-	-	[31]
*Spirulina*	PBS/*Spirulina* composites	The formulated *Spirulina* (varying from 15% to 50% loading) and PBS with and without PBS-g-MAH	-	60% protein (on dry weight basis)	[32]

**Table 4:** Summary of conducted studies: other species.

Biomass Species	Type of Product	Ratio of Materials	Characterization	Reference
*Chlorogloea fritschii*	bioplastic poly-3-hydroxybutyrate	-	PHB levels at 14–17% (*w*/*w* DW)	[33]
*Phaeodactylum tricornutum*	bioplastic PHB	-	PHB levels of up to 10.6% of algal dry weight	[11]
*Calothrix scytonemicola, Scenedesmus almeriensis and Neochloris oleoabundans,*	bio-based plastic film	1:2, Carboxymethyl Cellulose (CMC):biomass	-	[35]
*Calothrix scytonemicola*	PHA, plastic film	Product 1: 150 mg pure PH3B and 8 mL of chloroform	-	[35]
		Product 2: 100 mg of PH3B and 50 mg CMC mixed with 8 mL of CMC.		
		Product 3: 100 mg PH3B and 50 mg sucrose octa acetate in 8 mL of CMC		
*Nannocloropsis gaditana*	Bio composites: biomass and PBAT	Ratios of biomass: 10, 20, 30	-	[34]

**Table 5:** Blended materials with microalgae and used chemicals in the blending process.

Blended Materials with Biomass	Chemical Formula	Purpose of Usage	Reference
PE	(C_2_H_4_)_n_	Blended with *Chlorella* and *Spirulina*	[9,27]
PP	(C_3_H_6_)_n_	Blended with *Chlorella*	[25]
PVA	(C_2_H_4_)_n_	Blended with *Chlorella*	[25]
Wheat gluten		Blended with *Spirulina* platensis	[30]
PBS	(C_8_H_12_O_4_)_n_	Blended with *Spirulina*	[32]
UHMW-PE	C_2_H_4_	Blended with *Spirulina*	[31]
PVA-g-MAH (maleic anhydride-grafted PVA)		Used in blending process	[25]
Acetone	C_3_H_6_O	Used in blending process	[27,35]
Sodium sulfite	Na_2_SO_3_	Used in blending process	[32]
BPO	C_14_H_10_O_4_	Used in blending process	[22]

**Table 6:** Plasticizers and compatibilizers.

Plasticizers and Compatibilizers	Chemical Formula	Purpose of Usage	Reference
Glycerol	C_3_H_8_O_3_	Plasticizer	[22,26,29,30,32,50]
Octanoic acid	C_8_H_16_O_2_	Plasticizer	[30]
1,4-butanediol	C_4_H_10_O_2_	Plasticizer	[30]
EG	C_2_H_6_O_2_	Plasticizer	[31]
CMC		Plasticizer	[35]
MA	C_4_H_2_O_3_	Compatibilizer and grafting PVA	[22,25]
PE-g-MA		Compatibilizer	[31]
KPS	K_2_S_2_O_8_	Compatibilizer initiator	[22]
DMSO	(CH_3_)_2_SO	Compatibilizer initiator	[22]

**Table 7:** Other chemicals used in the process.

Other Chemicals Used in the Process	Chemical Formula	Purpose of Usage	Reference
Ethanol	C_2_H_5_OH	Suspension of biomass	[24]
IPP (isotactic polypropylene)	(C_3_H_6_)_n_	-	[25]
Citric acid	C_6_H_8_O_7_	Bioplastic film preparation	[26]
DCP (Dicumyl peroxide)	C_18_H_22_O_2_	In the synthesis of PBS-g-MAH	[32]
Methanol	CH_3_OH	To remove pigments in the PHB extraction process	[33]
CMC	CHCl_3_	PHB extraction from *Chlorella fritschii* biomass	[33]
Phosphate buffered saline	Cl_2_H_3_K_2_Na_3_O_8_P_2_	Cell washing	[11]
Sodium hypochlorite (aq)	NaClO	PHA extraction	[35]
Sucrose octa acetate	C_28_H_38_O_19_	Casting of plastic film	[35]

**Table 8:** Comparison between open and closed cultivation systems.

	Open Ponds	Closed PBR Systems
Advantages	Higher production volume possible Easier to clean More durable Economically advantageous	Higher cell densities and productivities Can be sterilized, prevent contamination Better control of culture conditions High mixing (vertical tube PBRs)
Disadvantages	Difficult to control culture conditions Low cell densities and productivities High contamination risk Restricted to hardy species	pH or oxygen gradients are a challenge (horizontal tube PBRs) Complex cleaning process Complex systems that might be expensive Difficult to scale up

**Table 9:** Summary of production methods to produce microalgae-polymer blend bioplastics.

Production Methods	Publications
Melt mixing	[22,23,27,32,84]
Compression molding	[9,27,30,31,32,84,86]
Hot molding	[22,23]
Injection molding	[34]
Twin screw extrusion	[34,87]
Solvent casting	[26,84,85,88]

**Table 10:** Summary of testing methods for microalgae–polymer blend bioplastics.

Testing Methods	Publications
Mechanical testing	[9,22,23,26,27,30,31,32,34,84,85,86,88,90]
Thermal based analysis (TGA, DSC)	[9,26,30,31,32,34,85,86]
SEM	[9,23,26,30,31,34,84,85,88]
CLSM	[34]
FT-IR	[26,27,31,32,34,85]
WAXS, SAXS, XRD	[84]
Wetting and water permeability	[30,84]
Oxygen permeability	[84]
Transparency	[84]
Odor panel test	[86]
